# Socioeconomic correlates of social asymmetry in English older adults: findings from a population-based cohort study

**DOI:** 10.1093/geronb/gbag195

**Published:** 2026-09-15

**Authors:** Bill Calvey, James Laurence, Joanna McHugh Power

**Affiliations:** Social Research Institute, University College London, London, United Kingdom; Social Research Institute, University College London, London, United Kingdom; Department of Psychology, Maynooth University, Maynooth, Ireland; (Psychological Sciences Section)

**Keywords:** Loneliness, Social isolation, Social vulnerability, Socioeconomic status, Cohort study design/analysis

## Abstract

**Objectives:**

Discrepancies between subjective loneliness and objective social isolation (i.e., social asymmetry) are linked to physical and psychological health in later life, but socioeconomic correlates of social asymmetry remain underexplored. We examined whether individual- and area-level socioeconomic indicators were longitudinally associated with social asymmetry in English older adults.

**Methods:**

Data were drawn from 8,164 adults (aged 60+) participating in Waves 7–9 (2014–2019) of the English Longitudinal Study on Ageing. Social asymmetry was operationalized as a continuous discrepancy score between standardized loneliness and social isolation, with positive values indicating social resilience (i.e., lower loneliness relative to isolation) and negative values indicating social vulnerability (i.e., higher loneliness relative to isolation). Time-varying generalized estimating equations assessed longitudinal associations between social asymmetry and socioeconomic indicators.

**Results:**

All socioeconomic indicators were associated with social asymmetry, even after adjusting for psychological covariates. Third-level education (*β* = 0.44, *p* < .001), higher household income (*β* = 0.22, *p* < .001), and subjective social status (*β* = 0.004, *p* < .001) were associated with social resilience. Conversely, individual- (*β* = −0.04, *p* < .001) and area-level deprivation (*β* = −0.20, *p* < .001), population density (*β* = −0.18, *p* < .001), rare/no internet use (*β* = −0.26, *p* < .001), unemployment (*β* = −0.07, *p* < .05), and renting in either private (*β* = −0.35, *p* < .001) or social housing (*β* = −0.41, *p* < .001) were associated with social vulnerability.

**Discussion:**

Addressing loneliness in later life may require interventions that target broader community-level socioeconomic inequalities and extend beyond individual behavioral change.

Social isolation refers to the objectively measurable absence or paucity of social contacts, commonly operationalized through indicators such as network size, frequency of interaction, and social participation (Cohen et al., 1997; Holt-Lunstad, 2018). In contrast, loneliness reflects a subjective sense of insufficiency or dissatisfaction with one’s social relationships, independent of their objective quantity (Hawkley & Cacioppo, 2010; Weiss, 1973). Although social isolation and loneliness tend to be moderately correlated, they are also conceptually distinct: individuals with fewer contacts tend, on average, to report greater loneliness, yet objective isolation accounts for only a portion of the variance in loneliness (Cornwell & Waite, 2009; Coyle & Dugan, 2012; de Jong Gierveld & Havens, 2004). This distinction is consequential, as both constructs are robustly associated with increased mortality, physical morbidity, cognitive function, and poorer psychological well-being (Evans et al., 2019; Holt-Lunstad et al., 2015; Park et al., 2020).

Early theoretical work emphasized that loneliness is multidimensional, comprising distinct emotional and social subtypes (Weiss, 1973). Emotional loneliness arises from the absence of a close attachment figure, whereas social loneliness reflects deficits in broader group belonging and social integration. This theoretical perspective highlights that loneliness emerges when there is a mismatch between the quality and/or quantity of social relationships individuals have and those they desire (Peplau & Perlman, 1982). For example, individuals with relatively rich social networks may still experience loneliness if these network ties fail to provide adequate support or meaning (Newall & Menec, 2019), while others may be satisfied with their limited social circle and report low levels of loneliness as a result. Consequently, loneliness and social isolation can diverge in important ways, underscoring the need to examine them jointly rather than as interchangeable constructs (Cornwell & Waite, 2009; van Tilburg, 2025).

Building on these distinctions between objective and subjective social connection, a *social asymmetry* framework was recently developed to capture the mismatch between objective social isolation and subjective feelings of loneliness (McHugh Power et al., 2017; Ong et al., 2024). Prior research identified four isolation–loneliness profiles: (a) isolated and lonely, (b) isolated but not lonely, (c) not isolated but lonely, and (d) neither isolated nor lonely (McHugh Power et al., 2017). Individuals whose isolation and loneliness levels are aligned are considered *concordant*, whereas those exhibiting discrepancies are *discordant*. Discordant profiles may reflect either *social vulnerability* (i.e., experiencing higher loneliness than levels of isolation) or *social resilience* (i.e., experiencing lower loneliness than levels of social isolation). We caution against adopting an overly narrow interpretation of social resilience as operationalized in this and other recent studies (e.g., Jiang & Yu, 2025; Ong et al., 2025). Such patterns, where loneliness is lower than isolation, may also reflect active psychological adaptation, a preference for solitude or differing social expectations, rather than resilience per se.

While social asymmetry is a relatively new construct (McHugh Power et al., 2017; Ward et al., 2021), emerging evidence suggests that it has important implications for health and aging. Both *discordant* profiles (i.e., social resilience and social vulnerability) are linked to elevated mortality risk compared to individuals who are neither isolated nor lonely (Ward et al., 2021), with effects varying by sex and cause of death. Beyond survival, social vulnerability is associated with poorer physical health in varying cultural contexts (Ong et al., 2025). Socially vulnerable older adults also experience steeper cognitive decline, have an elevated risk of developing dementia, and experience more psychological distress (Menec et al., 2019; Ng et al., 2025), whereas socially resilient older adults generally perform better in memory, executive functioning, and global cognition (McHugh Power et al., 2017; Power et al., 2019).

The significance of social asymmetry for health and wellbeing in later life is increasingly recognized, making it essential to understand what shapes older adults’ experiences of it. However, research on the antecedents of social asymmetry remains limited, with most studies focusing on psychological and health-related correlates. For example, Jiang and Yu (2025) identified depression, anxiety, stress, and sleep disturbances as key correlates of social asymmetry, while Miura et al. (2024) found that fewer depressive symptoms and higher extraversion were associated with greater social resilience. Socioeconomic factors may also play a role: education, income, retirement status, functional disability, and multimorbidity have been shown to relate differently to social isolation and loneliness among Japanese older adults (Kino et al., 2023), hinting that socioeconomic status (SES) may also be key in predicting social asymmetry. Yet these existing studies have largely been conducted in East Asian contexts and have cross-sectionally examined a narrow set of individual- and area-level SES indicators. As a result, this represents a critical gap in the literature, as there is limited understanding of how broader social structures, particularly SES, shape social asymmetry in older populations, especially over time as social networks have the propensity to decline (Wrzus et al., 2013).

SES is robustly associated with both social isolation and loneliness in older adults (Courtin & Knapp, 2017; Finlay & Kobayashi, 2018), but SES may be particularly salient for social asymmetry. Theoretical perspectives propose that loneliness emerges not simply from the number of social ties individuals possess, but from how existing relationships compare with their expectations and relational needs (Peplau & Perlman, 1982). SES may therefore shape the extent to which objectively similar levels of social contact are experienced as sufficient and meaningful. Older adults with greater socioeconomic resources may possess more capacity to maintain valued relationships, participate in meaningful social roles, and exert control over their social environments, buffering against loneliness even in the context of relative isolation. Conversely, socioeconomic disadvantage may heighten stress exposure, perceived exclusion, and unmet relational expectations, increasing vulnerability to loneliness despite comparable levels of social contact. Contemporary perspectives increasingly position loneliness as being socially patterned by broader structural inequalities rather than solely individual-level factors (Nyqvist et al., 2025), positioning itself as a social justice issue (Barreto et al., 2024). From this perspective, SES is likely to shape social asymmetry status in later life as it influences both opportunities for social connection and the subjective meanings older adults attach to relationships (Engstrom et al., 2024).

We therefore propose that SES in later life may shape how far given levels of objective social connection are translated into felt connection, consequently affecting individuals’ social asymmetry. In particular, we hypothesize that lower SES may increase social vulnerability, whereas higher SES may be associated with greater social resilience. At the individual-level, socioeconomic disadvantage may influence the extent to which existing relationships meet the needs of older adults (Peplau & Perlman, 1982). Indicators such as lower income, retirement status, or greater material deprivation may heighten exposure to financial insecurity, which in turn might prohibit older adults from being satisfied with preexisting relationships (Barjaková et al., 2023; Kino et al., 2023; Mangot-Sala et al., 2025; Wu et al., 2022). For example, low income or material deprivation may limit ability to participate in shared activities or may increase feelings of burden. Other individual-level indicators of SES may be important for social asymmetry such as internet use, housing tenure and subjective social status. Access to, and use of, the internet may enable maintenance of geographically dispersed ties or support those with limited mobility. Meanwhile housing tenure may further shape residential stability and local social embeddedness, potentially influencing feelings of security and neighborhood cohesion (Carbone et al., 2022; Morris & Verdasco, 2021). Furthermore, lower subjective status may intensify feelings of marginality or exclusion (Barjaková et al., 2023; Wu et al., 2022), particularly during later-life transitions such as retirement, declining income, or functional decline. Such feelings of marginality may heighten social asymmetry by reducing individuals’ perceived social value or belonging, even when their actual social contact remains unchanged.

Beyond individual resources, area-level socioeconomic conditions may also be relevant to social asymmetry because they shape the local contexts in which later-life relationships are maintained and experienced. For older adults, who are often more reliant on their immediate neighborhoods, living in deprived or densely populated areas may limit the extent to which social contact is experienced as stable, supportive, and meaningful. In particular, geographic factors such as area-level deprivation and urbanity are associated with loneliness and social isolation among older adults (Chen et al., 2024; Finlay & Kobayashi, 2018; Menec et al., 2019; Röhr et al., 2022) and may limit opportunities for stable, trusting, and reciprocal ties, such that contact does not readily translate into felt belonging. Higher population density, characteristic of more urban areas, may also be important for social asymmetry. While it likely increases opportunities for contact, such contact is often more superficial in denser, urban areas; local belonging is often more tenuous; while high residential turnover and transience in such areas may limit the development of stable, enduring and meaningful local ties (Lai et al., 2021). Taken together, individual- and area- level dimensions of SES may shape social asymmetry in later life via multiple, overlapping pathways.

Psychosocial factors may also play an important role in shaping the relationship between social asymmetry and SES by influencing how individuals evaluate and emotionally respond to their social relationships. Depression is intrinsically linked with isolation, loneliness, and also social asymmetry as a consequence (Ge et al., 2017; Jiang & Yu, 2025). Higher depressive symptoms may also heighten social cognitions, sensitivity to rejection and perceptions of interpersonal inadequacy (Gao et al., 2017; Santini et al., 2020), thereby increasing loneliness even when objective social contact is maintained. Self-rated health may also be relevant, given that both isolation and loneliness are linked to health in later life (Cacioppo & Hawkley, 2003) and that poorer health status has the capacity to restrict social participation (Griffith et al., 2017). Additionally, life satisfaction reflects broader evaluations of whether emotional and social needs are being fulfilled and may therefore capture dimensions of subjective wellbeing beyond depression or psychological distress alone (Mansfield et al., 2026). Recent evidence suggests that social isolation is associated with poorer self-rated health, lower life satisfaction and greater psychological distress in later life, with these associations potentially accumulating across multiple domains of wellbeing (Mansfield et al., 2026). Given that SES is closely linked to these psychosocial factors, and that psychosocial factors are strongly related to social asymmetry, these factors may attenuate the association between social asymmetry and SES in later life.

The present study therefore aims to address prevailing gaps within the literature by examining whether individual- and area-level indicators of SES are longitudinally associated with social asymmetry in a sample of English older adults. Specifically, we focus on two manifestations of social asymmetry on one continuous scale: loneliness that exceeds levels of objective isolation (i.e., social vulnerability) and social isolation that exceeds levels of loneliness (i.e., social resilience). In addition, we will explore how the associations between indicators of SES and social asymmetry may be attenuated by potential psychosocial factors associated with social asymmetry, such as depression, self-rated health and satisfaction with life.

## Method

### Study design and population

The present study used archived secondary data from the English Longitudinal Study of Ageing (ELSA) to address the study objectives. ELSA is an ongoing, multiwave prospective cohort study of adults aged 50 and older years living in England, designed to capture longitudinal measurements of multidimensional aspects of health, occupation, economic status and retirement (Steptoe et al., 2013). Participants were eligible for inclusion in ELSA if: (a) they had participated in the Health Survey for England (HSE) in 1998, 1999, and 2001 and agreed to follow-up; (b) were born before March 1, 1952; and (c) lived in a private household in England at baseline (2002/2003). ELSA data are collected biennially, with 11 waves of ELSA data currently archived (https://doi.org/10.5255/UKDA-Series-200011). Baseline assessments consisted of electronic, self-reported questionnaires, while nurse-led interviews collecting physical and medical data were conducted every two waves, starting at Wave 2. Participants provided written informed consent prior to data collection. Area-level socioeconomic indicators used in the analyses were accessed under Special Licence conditions, through the UK Data Service.

We analyzed data from ELSA Waves 7 (2014/2015), 8 (2016/2017), and 9 (2018/2019). Later ELSA waves were selected due to the availability of comprehensive socioeconomic indicators potentially relevant to social asymmetry. The most recent waves were excluded to ensure that social asymmetry and its predictors were not influenced by the COVID-19 pandemic, which began to cross over during ELSA Wave 10. We excluded participants who were younger than 60 years old at any point during the study period, consistent with prior social asymmetry studies in older adults (McHugh Power et al., 2017; Power et al., 2019). Additionally, we excluded participants who completed a partial or proxy interview at any point during the study period (*n* = 763), to avoid the analysis of proxy-reported responses. Ultimately, 8,164 unique participants contributed to our analyses (Wave 7: *n* = 6,965; Wave 8: *n* = 6,720; Wave 9: *n* = 6,350).

### Measures

#### Outcome: social asymmetry

Social Asymmetry quantifies the discrepancy between subjective loneliness scores and objective social connectedness. In ELSA, loneliness scores were measured using the three-item UCLA Loneliness Scale (Russell, 1996), yielding scores ranging from 3 to 9. We rescaled these UCLA loneliness scores by subtracting 3 from each score, resulting in a range from 0 to 6, with higher scores indicating greater loneliness. Objective social isolation was measured using a modified version of the Berkman-Syme Social Network Index (SNI) (Berkman & Syme, 1979). Although the SNI was not directly administered in ELSA, sufficient information was available to reconstruct it, as done previously (McHugh Power et al., 2017). The SNI included indicators of marital or cohabiting status, presence of close friends and membership in social or community groups. One modification was required, whereby the original item assessing attendance at religious services was not captured in ELSA and, as a result, was replaced with an item capturing membership in a religious organization. The modified SNI yielded scores ranging from 0 to 4, based on whether participants were integrated (=0) or isolated (=1) for each of the four items, with higher scores indicating higher social isolation. Table S1 tabulates the various items used to construct our measures of loneliness and social isolation.

Social asymmetry was calculated by converting both loneliness and isolation scores into *Z* scores, standardizing both scales within each of the three ELSA waves. Loneliness scores were then subtracted from social isolation scores, which yielded a continuous discrepancy score for each participant (Calvey et al., 2024a, 2024b; McHugh Power et al., 2017). Positive discrepancy scores indicate “social resilience,” whereby individuals report lower loneliness than their level of social isolation. Negative discrepancy scores indicate social vulnerability, reflecting higher loneliness than their social isolation. Scores close to zero indicate concordance between subjective loneliness and objective social isolation.

Prior work has operationalized social asymmetry using both categorical typologies (e.g., McHugh Power et al., 2017) and residualized approaches (e.g., Ong et al., 2025), each of which captures different conceptualizations of the relationship between loneliness and isolation. We adopted a standardized discrepancy score approach because the aim of this study was to operationalize social asymmetry as the relative discrepancy or distance between subjective loneliness and objective isolation, rather than to model loneliness as an outcome predicted by isolation. This approach provides a direct measure of the divergence between the two constructs, with both variables first placed on a common metric via standardization prior to computing the discrepancy score. This ensures that loneliness and isolation contribute equally to the resulting asymmetry measure despite being assessed using different instruments and response scales. In contrast to model-dependent residuals, which represent deviation from a predicted value conditional on the sample-specific association between variables, the standardized difference-score approach offers a more transparent and directly interpretable representation of subjective–objective mismatch.

#### Exposures: socioeconomic indicators

Time-varying socioeconomic indicators were measured at each of the three ELSA waves of interest and captured SES at both an individual (education level, employment status, subjective social status, individual deprivation, household income, internet use and housing tenure) and area-level (area deprivation and population density). Education levels were ascertained from ISCED-97 classifications and grouped into four categories: none/primary, lower secondary, upper secondary, and third level education. Employment status was self-reported and categorized into three groups: “Retired,” “Employed,” and “Unemployed.” Subjective social status was assessed using a self-placement ladder ranging from 5 (*worst off*) to 100 (*best off*). Individual deprivation was assessed using a nine-item index capturing whether financial constraints prevented participants from engaging in common activities (e.g., hosting friends or family for meals or affording suitable clothing). Scores ranged from 0 to 9, with higher scores indicating greater material deprivation. Equivalized household income was categorized into quintiles, with quintile one representing the lowest income group. Internet use was self-reported by participants based on how regularly they used the internet: “Regularly,” “Sometimes,” or “Rarely/Never.” Housing tenure was also self-reported, whereby participants indicated if they were homeowners, renting from a private landlord, renting in social housing, or others (e.g., living rent-free). Area deprivation was measured using an Index of Multiple Deprivation captured at the Local Authority level. Local authorities were categorized into quintiles, with higher quintiles reflecting greater area-level deprivation. Finally, a measure of population density categorized participants into five quintiles of density, with higher quintiles reflecting living in a denser area than lower quintiles, also measured at the Local Authority level.

#### Psychosocial wellbeing

We also adjust model estimates for three indicators of psychological characteristics and wellbeing that are potentially linked to socioeconomic circumstances, loneliness and social isolation, and may act as psychosocial pathways between SES and social asymmetry: depressive symptoms, self-rated health and satisfaction with life. Depressive symptoms were measured using the eight-item Centre for Epidemiological Studies Depression (CES-D) scale (Radloff, 1977), with dichotomous responses summed to produce scores ranging from 0 to 8. However, since the CES-D contains an item relating to loneliness, we removed this item and calculated a seven-item CES-D score instead. Self-rated health was assessed at each wave using a single-item measure (Would you say your health is excellent, very good, good, fair or poor?), coded from 1 (*excellent*) to 5 (*poor*). We recoded these scores so that higher self-rated health scores indicated better perceived health. Finally, life satisfaction was assessed using the Satisfaction with Life Scale (Diener et al., 1985), comprising five items rated on a 7-point scale. Mean scores ranged from 1 to 7, with higher values indicating greater life satisfaction.

### Statistical analysis

All analyses were conducted in RStudio. There were relatively few missing datapoints in the dataset (7.19%). Little’s Missing Completely at Random test (Little, 1988) indicated that the missing data were unlikely to be missing completely at random (*p* < .001). Patterns of missingness were explored using the *naniar* R package (Tierney & Cook, 2023), which indicated that clusters of missingness appeared primarily in individual-level deprivation, subjective social status, and some items within the modified-SNI variables. Missingness in these variables was explained by our other covariates and missing variables (age, sex, and self-rated health), consistent with the “missing at random” mechanism.

Therefore, we imputed this missingness using the R package *Multiple Imputation by Chained Equations* (Van Buuren & Groothuis-Oudshoorn, 2011). Continuous data were imputed using predictive mean matching while categorical data were imputed via polytomous or logistic regression, where relevant. Following current methodological recommendations to use a sufficient number of imputations (i.e., a number greater than the overall % of missingness) to reduce Monte Carlo error and improve efficiency (e.g., Blomberg & Todorov, 2025), 20 imputed datasets were generated with 20 iterations of the imputation algorithm, with results combined into one final imputed dataset using Rubin’s rules.

Associations between SES and social asymmetry were examined using generalized estimating equations (GEE). GEE models estimate population-average effects while accounting for within-person correlation arising from repeated measurements across study waves. Social asymmetry was modeled as a continuous outcome using a Gaussian distribution with an identity link. Given the small number of equally spaced measurement occasions (i.e., three waves, two years apart), an exchangeable working correlation structure was specified, assuming constant within-person correlation over time. Previous studies have similarly applied linear GEE models to continuous loneliness and social isolation outcomes (Maas et al., 2025; Wang et al., 2019). GEE models can also achieve adequate statistical power with substantially smaller longitudinal samples than those used in the present study, even under moderate attrition and within-subject correlation assumptions (Liu & Liang, 1997).

A stepwise modeling approach was adopted. Firstly, each socioeconomic indicator was modeled separately, adjusting for time, age, sex and ethnicity (Step 1). Next, all socioeconomic indicators were included simultaneously in the same model, while adjusting for the same covariates to explore independence of the socioeconomic indicators (Step 2). Finally, psychosocial wellbeing variables (depressive symptoms, self-rated health and life satisfaction) were added to examine whether these factors attenuated associations between socioeconomic indicators and social asymmetry (Step 3).

We conducted additional sensitivity analyses to assess the robustness of our findings. We ran time-lagged GEE models in which socioeconomic indicators measured at time *t* predicted social asymmetry at time *t* + 1, thereby strengthening temporal ordering (see Table S2). We also repeated the main GEE analysis using the cross-sectional interview weights supplied by ELSA for each wave (see Table S3). These weights adjust for sampling design, differential non-response, and population representativeness. Given that the geeglm() function treats weights as precision weights rather than probability weights, this weighted model serves as a sensitivity analysis rather than a full survey-design correction. This study was not pre-registered. All code necessary to reproduce the analyses is provided in Supplementary Methods. All analyses were conducted in R using the *geepack* package (Højsgaard et al., 2006).

## Results

In total, 8,164 older adults from ELSA Waves 7 to 9 were included in the analyses. At baseline (i.e., ELSA Wave 7), the analytic sample comprised 6,965 participants, with a mean age of 71.2 years (±8.3 years). Just over half of the participants were women (54.4%), with the sample being predominantly White (97.0%). Most participants were retired (74.3%), with smaller proportions being employed (17.7%) or unemployed (7.9%). Educational attainment was mixed, with 23.8% having Primary or No education and 36.2% having obtained a third-level degree. The majority of the sample were homeowners (84.6%), with the remaining participants residing in private renting (2.6%), social housing (11.6%), and other housing (1.2%) conditions. Mean levels of depressive symptoms were low (Mean = 1.23 ± 1.65), self-rated health was moderate (Mean = 3.31 ± 1.09), and satisfaction with life score was relatively high (Mean = 22.9 ± 9.91). The average loneliness score was 1.07 (±1.48), and the mean social isolation score was 2.11 (±0.68). Full descriptives are presented in Table S5. Although some categories (e.g., within ethnicity and housing tenure variables) were less prevalent, all included predictors contained sufficient observations for reliable estimation, with no evidence that any sparse cells would compromise model convergence or stability. The large overall sample size (*n* = 8,164) and longitudinal structure further support the stability of GEE-based inference under asymptotic conditions (Liu & Liang, 1997).

**Table 1:** A set of sequential generalized estimating equation models testing the association between socioeconomic variables and social asymmetry as an outcome, among English older adults (*n* = 8,164).

Variables	Step 1				Step 2				Step 3			
	Estimate	*SE*	95% CI	*p*	Estimate	*SE*	95% CI	*p*	Estimate	*SE*	95% CI	*p*
**Time**	^a^	^a^	^a^		−0.03***	0.01	−0.05, −0.01	<.001	−0.03**	0.01	−0.04, −0.01	.002
**Age**	^a^	^a^	^a^		−0.01***	0.001	−0.01, −0.01	<.001	−0.003	0.003	−0.007, 0.001	.07
**Sex: female**	^a^	^a^	^a^		−0.09**	0.03	−0.15, −0.03	.002	−0.04	0.03	−0.09, 0.01	.11
**Ethnicity: non-White**	^a^	^a^	^a^		−0.10	−0.08	−0.23, 0.06	.22	−0.12	0.07	−0.27, 0.02	.09
**Individual-level SES**												
**Education**												
Lower secondary	0.48***	0.04	0.39, 0.57	<.001	0.27***	0.06	0.13, 0.29	<.001	0.24***	0.05	0.16, 0.31	<.001
Upper secondary	0.64***	0.06	0.52, 0.76	<.001	0.21***	0.04	0.15, 0.38	<.001	0.32***	0.04	0.21, 0.42	<.001
Third level	0.96***	0.04	0.88, 1.05	<.001	0.41***	0.04	0.33, 0.50	<.001	0.44***	0.04	0.36, 0.51	<.001
**Employment status**												
Employed	−0.04	0.03	−0.10, 0.02	.15	−0.11***	0.03	−0.16, −0.05	<.001	−0.09**	0.03	−0.14, −0.04	<.001
Unemployed	−0.29***	0.04	−0.37, −0.22	<.001	−0.17**	0.04	−0.24, −0.10	<.001	−0.07*	0.03	−0.14, −0.01	.03
**Subjective social status**	0.017***	0.001	0.016, 0.019	<.001	0.01***	0.001	0.009, 0.012	<.001	0.004***	0.001	0.002, 0.005	<.001
**Individual deprivation**	−0.14***	0.01	−0.15, −0.12	<.001	−0.09***	0.01	−0.11, −0.08	<.001	−0.04***	0.01	0.06, −0.03	<.001
**Household income**												
Q2	0.16***	0.03	0.09, 0.23	<.001	0.07*	0.03	0.01, 0.13	.04	0.08*	0.03	0.02, 0.14	.01
Q3	0.35***	0.04	0.28, 0.42	<.001	0.17***	0.04	0.10, 0.23	<.001	0.16***	0.03	0.10, 0.22	<.001
Q4	0.51***	0.04	0.43, 0.58	<.001	0.20***	0.04	0.13, 0.27	<.001	0.18***	0.03	0.12, 0.24	<.001
Q5 (highest)	0.65***	0.04	0.57, 0.72	<.001	0.26***	0.04	0.19, 0.33	<.001	0.22***	0.03	0.16, 0.29	<.001
**Internet use**												
Sometimes	−0.18***	0.02	−0.23, −0.12	<.001	−0.07*	0.03	−0.21, −0.06	.01	−0.03	0.03	−0.08, 0.02	.23
Rarely, never	−0.59***	0.03	−0.65, −0.52	<.001	−0.32***	0.03	−0.37, −0.23	<.001	−0.26***	0.03	−0.32, −0.20	<.001
**Housing tenure**												
Private renter	−0.62***	0.09	−0.80, −0.43	<.001	−0.36***	0.09	−0.53, −0.19	<.001	−0.27***	0.08	−0.43, −0.12	<.001
Social housing renter	−1.09***	0.05	−1.20, −0.99	<.001	−0.56***	0.05	−0.67, −0.45	<.001	−0.41***	0.05	−0.51, −0.32	<.001
Other	−0.36**	0.11	−0.70, −0.20	.002	−0.23*	0.11	−0.44, −0.01	.04	−0.18	0.11	−0.39, 0.03	.09
**Area-level SES**												
**Area deprivation**												
Q2	−0.10**	0.03	−0.16, −0.04	.002	−0.03	0.03	−0.09, 0.04	.40	0.02	0.03	−0.07, 0.04	.56
Q3	−0.20***	0.04	−0.27, −0.12	<.001	−0.03	0.04	−0.09, 0.04	.46	−0.04	0.03	−0.10, 0.03	.25
Q4	−0.44***	0.04	−0.53, −0.36	<.001	−0.09*	0.04	−0.17, −0.01	.03	−0.06	0.04	−0.14, 0.01	.10
Q5 (highest)	−0.84***	0.05	−0.94, −0.74	<.001	−0.25***	0.05	0.35, −0.15	<.001	−0.20***	0.05	−0.29, −0.10	<.001
**Population density**												
Q2	−0.16***	0.04	−0.23, −0.09	<.001	−0.10**	0.04	−0.17, −0.03	.004	−0.08*	0.03	−0.14, −0.01	.02
Q3	−0.28***	0.04	−0.36, −0.21	<.001	−0.15***	0.04	−0.22, −0.07	<.001	−0.11**	0.04	−0.17, −0.04	.001
Q4	−0.36***	0.04	−0.44, −0.27	<.001	−0.17***	0.04	−0.24, −0.09	<.001	−0.12***	0.04	−0.19, −0.04	<.001
Q5 (greatest density)	−0.49***	0.05	−0.59, −0.39	<.001	−0.21***	0.05	−0.31, −0.12	<.001	−0.18***	0.04	−0.27, −0.10	<.001
**Psychosocial confounders**												
**Depressive symptoms**									−0.12***	0.01	−0.14, −0.11	<.001
**Self-rated health**									0.03***	0.01	0.01, 0.05	<.001
**Satisfaction with life**									0.07***	0.002	0.06, 0.07	<.001

*Note. SE* = standard error; SES = socioeconomic status; 95% CI = 95% confidence interval.

a Step 1 models were adjusted for time, age, sex, and ethnicity, but estimates were not reported for these controls in Step 1. Reference categories: sex: male; ethnicity: White; education: none/primary level; employment status: retired; area deprivation: Quintile 1 (lowest deprivation); household income: Quintile 1 (lowest earners); population density: Quintile 1 (lowest density); internet use: regularly; housing tenure: homeowner.

* *p* < .05.

** *p* < .01.

*** *p* < .001.

Initially, each socioeconomic indicator was entered into a separate GEE model (Table 1) alongside time, age, sex, and ethnicity, providing minimally adjusted estimates of their associations (Step 1). Higher educational attainment was associated with social asymmetry in the direction of social resilience (where an individual’s loneliness was lower than their social isolation), with progressively larger estimates for upper secondary (*β* = 0.64, 95% CI [0.52, 0.76]) and third-level education (*β* = 0.96, 95% CI [0.88, 1.05]) relative to those with no or primary education. Higher household income was similarly associated with social resilience, with individuals in the highest income quintile (*β* = 0.65, 95% CI [0.57, 0.72]) showing higher resilience compared with those in the lowest quintile. Higher subjective social status was associated with social resilience (*β* = 0.017, 95% CI [0.016, 0.019]), while greater individual deprivation was associated with social vulnerability (*β* = −0.14, 95% CI [−0.15, −.012]), where an older adult’s loneliness was greater than their social isolation score. Indicators of material disadvantage were consistently associated with social vulnerability. Despite a small number of participants within these categories, private renters (*β* = −0.62, 95% CI [−0.80, −0.43]) and social housing renters (*β* = −1.09, 95% CI [−1.20, −0.99]) were significantly more socially vulnerable, compared to homeowners. Less frequent internet use was also associated with social vulnerability, particularly among those who rarely or never used the internet (*β* = −0.59, 95% CI [−0.65, −0.52]). At the area level, area deprivation and population density were increasingly associated with social vulnerability, with individuals in the highest quintiles of area deprivation (*β* = −0.84, 95% CI [−0.94, −0.74]) and population density (*β* = −0.49, 95% CI [−0.59, −0.39]) being significantly more socially vulnerable than those in the lowest quintiles respectively.

In Step 2, all individual- and area-level socioeconomic indicators were entered simultaneously, while still adjusting for time, age, sex, and ethnicity. Following this additional adjustment, the strength of the estimates for several socioeconomic indicators, including education, household income, individual deprivation, housing tenure, internet use, and area deprivation, was reduced, indicating shared variance across socioeconomic dimensions. For example, third-level education was still significantly associated with social asymmetry after adjustment for other socioeconomic indicators (*β* = 0.41, 95% CI [0.33, 0.50]), but to a lesser extent in Step 1 (*β* = 0.96, 95% CI [0.88, 1.05]). Similarly, the strength of the association between the highest quintile of area deprivation and social asymmetry (*β* = −0.84, 95% CI [−0.94, −0.74]) was reduced after adjusting for other socioeconomic indicators (*β* = −0.25, 95% CI [−0.35, −0.15]). However, each indicator remained independently associated with social asymmetry even when modeled collectively.

In Step 3, psychosocial factors were added to an attenuated socioeconomic model. Increasing depressive symptoms were strongly associated with social vulnerability (*β* = −0.12, 95% CI [−0.14, −0.11]), whereas better self-rated health (*β* = 0.03, 95% CI [0.01, 0.05]) and greater satisfaction with life (*β* = 0.07, 95% CI [0.07, 0.07]) were significantly associated with social resilience. Inclusion of these psychosocial variables led to further attenuation of several socioeconomic associations, in particular individual deprivation (*β* = −0.04, 95% CI [0.06, 0.03]) where its association with social asymmetry weakened. Sex was no longer significantly associated with social asymmetry once psychosocial confounders were introduced. However, higher educational attainment, higher household income, subjective social status, individual-level deprivation, housing tenure, internet use, and area disadvantage/density indicators remained independently associated with social asymmetry in the attenuation model.

As a sensitivity analysis, we also ran a time-lagged GEE model, whereby SES factors at time *t* predicted social asymmetry scores at time *t* + 1, among a sample of *n* = 6,586 older adults. Overall, the findings largely mirrored those of the main contemporaneous analyses, indicating that socioeconomic associations in social asymmetry persist over time and are not solely attributable to cross-sectional associations. Some minor discrepancies did arise between the two analyses. Being unemployed was not associated with future social asymmetry in the time-lagged analysis, relative to being retired. Household income was more strongly associated with future social asymmetry scores in the time-lagged analysis (*β*  =  0.30, 95% CI [0.21, 0.39]) than concurrent social asymmetry in the main analysis (*β*  =  0.22, 95% CI [0.16, 0.29]). Belonging to the “Other” housing category was not significantly associated with future social asymmetry scores in the supplementary analysis, compared to homeowners. Finally, those who accessed the internet sometimes were not significantly different from regular users in terms of their association with future social asymmetry scores. We also conducted a sensitivity analysis, which included ELSA weights to adjust for sample representativeness and attrition. This analysis produced similar estimates to those found in our main models (see Table S3). One notable discrepancy was that reporting internet use “sometimes” (relative to regularly) was significantly associated with social vulnerability (*β* = −0.06, 95% CI [−0.12, −0.002]), while this finding was not statistically significant in the main model.

## Discussion

While prior work has largely conceptualized social asymmetry as a function of individual psychological characteristics, our study demonstrates that broader socioeconomic stratification may also play a central role in shaping whether social connections translate into feelings of loneliness or belonging. Drawing on three consecutive waves of ELSA, we found that social asymmetry is socioeconomically patterned in later life, among English older adults. Indicators of socioeconomic advantage were significantly associated with social resilience (i.e., where objective isolation exceeds subjective loneliness), whereas indicators of socioeconomic disadvantage were generally associated with social vulnerability (i.e., where subjective loneliness exceeds objective isolation).

These findings extend the limited evidence base demonstrating that education, income, and retirement status are discordantly associated with loneliness and isolation (e.g., Kino et al., 2023) by incorporating a wider range of individual- and area-level socioeconomic indicators, such as material deprivation, population density, housing tenure, internet use, and subjective social status, to test their association with social asymmetry directly. Although effect sizes were attenuated after adjusting for key psychological and wellbeing characteristics, all socioeconomic factors remained independently associated with social asymmetry, even when a time-lagged modeling approach was adopted. Together, these results suggest that social asymmetry is not solely an intrapersonal phenomenon, but reflects structured inequalities in resources, status and lived environments.

Several different pathways may plausibly explain why SES is associated with social asymmetry. One explanation lies in the stress process model (Pearlin et al., 1981), which posits that chronic strain undermines coping resources, reduces perceived control, and heightens perceived threat. Here, material resources (such as income and deprivation) and housing security may contribute to psychological strain. In particular, financial hardship may introduce chronic environmental stress that alters how social interactions are experienced. Under the weight of these strains, social ties may feel less reassuring, everyday interactions more emotionally taxing, and support less accessible (Hawkley & Cacioppo, 2010). In this context, objective social contact may fail to translate into subjective connection, as strained psychological resources erode the emotional benefits typically derived from relationships, ultimately driving social vulnerability. Conversely, socioeconomic advantage may protect against such strain. Greater economic security (in the form of higher income, lower deprivation and stable housing) may buffer exposure to these chronic stressors, preserving the psychological agency needed to convert objective contact into felt belonging.

A second, related mechanism concerns the structural capacity to satisfy relational needs, where loneliness arises, in part, when there is a discrepancy between desired and actual support (Peplau & Perlman, 1982). Rather than operating purely through psychological strain, factors like digital access and area context condition the physical feasibility of sustaining relationships. Limited digital access may restrict an older adult’s capacity to maintain geographically dispersed networks or engage in modern, remote social connectivity, effectively cutting off pathways to support when physical mobility declines and potentially limiting the emotional or practical support available within existing ties (Cohen & Wills, 1985).

Area context may dictate the availability and safety of local “third places” and community infrastructure. Higher population density was associated with greater social vulnerability; urban environments may increase opportunities for social contact while simultaneously weakening neighborhood cohesion and relational depth. Area deprivation may further constrain opportunities for social participation through reduced access to safe, accessible community infrastructure and social spaces. Consequently, even if an individual’s objective network size remains stable, a lack of digital and local scaffolding reduces opportunities to actively invest in and maintain those relationships (Janssen et al., 2025; van Tilburg, 2025). This may widen the gap between relational provision and desire, driving social vulnerability despite comparable levels of baseline contact. At the same time, these mechanisms are unlikely to operate independently or exclusively. Structural factors, such as housing conditions, digital exclusion, and neighborhood deprivation, may also influence psychological resources, perceived control, and stress exposure, suggesting that material, environmental, and psychosocial pathways likely overlap in shaping social asymmetry in later life.

These findings carry important implications for policy moving forward. Interventions targeting loneliness in later life have often focused on increasing social contact or enhancing individual coping skills and resilience (Paquet et al., 2023; van Tilburg, 2025). While these approaches can be valuable, they may be insufficient if structural conditions shaping how relationships are formed and experienced remain unaddressed (Nyqvist et al., 2025). Socioeconomic disadvantage appears to heighten social vulnerability, indicating that material deprivation, digital exclusion, insecure housing and limited access to community resources may constrain older adults’ capacity to translate social contact into meaningful felt belonging. Policies aimed at reducing loneliness could therefore extend beyond individual-level interventions to include investments in digital inclusion, housing stability and age-friendly community infrastructure (Shekelle et al., 2024). Addressing social vulnerability requires not only creating opportunities for contact but also reducing the structural inequalities that may help explain whether those contacts are experienced as supportive, valued and socially integrating.

Importantly, the relationship between SES and social asymmetry in later life may be particularly consequential due to the distinct social and structural transitions that characterize aging. Older adulthood is often marked by contraction of social networks driven by retirement, bereavement and declining physical health (Wrzus et al., 2013). As networks have the propensity to shrink, older adults may shift their socioemotional goals, placing a greater emphasis on the emotional quality and adequacy of their remaining relationships rather than network size (Carstensen et al., 1999). In this context, socioeconomic resources may become particularly important to social asymmetry, as material security, housing stability, and digital access can support the maintenance of meaningful social connections despite age-related constraints. Conversely, socioeconomic disadvantage may amplify the impact of physical limitations and area-level barriers that restrict opportunities for social participation. Our findings therefore suggest that in later life, objective social contact may be more likely to translate into subjective belonging when sufficient structural and material resources are present to sustain social engagement.

More broadly, our study contributes to a growing body of research that examines social asymmetry and its relationship with health and aging. Discrepancies between objective isolation and subjective loneliness have been longitudinally associated with survival, cognitive decline and physical health (McHugh Power et al., 2017; Ong et al., 2025; Ward et al., 2021), underscoring the prognostic significance of how older adults experience their social worlds. However, much of this literature has focused on psychological or health-related outcomes, with comparatively less attention drawn to the structural conditions that shape these discrepancies in the first place. Our study offers valuable insights into the structural and socioeconomic context that conditions older adults to skew toward social vulnerability or resilience. At the same time, social asymmetry remains a concept undergoing active refinement. It has been operationalized using both categorical 2 × 2 typologies (McHugh Power et al., 2017) and regression-based residual approaches (Jiang & Yu, 2025; Ong et al., 2025), reflecting ongoing theoretical and methodological development within the field. Despite this heterogeneity, a consistent pattern is emerging: discrepancies between subjective experience and objective social conditions carry meaningful implications for later-life health and wellbeing.

However, there are several limitations to our study design that warrant acknowledgement. First, measurement inconsistencies across ELSA waves constrained the range of variables available for analysis and limited the number of waves that could be included, thereby reducing follow-up duration. In particular, we were unable to incorporate a broader set of psychological characteristics (e.g., personality traits or sleep quality) previously linked to social asymmetry (Jiang & Yu, 2025). We also excluded the most recent waves to avoid potential confounding associated with the COVID-19 pandemic, which substantially altered patterns of social contact and loneliness. Although area-level deprivation and population density measures were included in the analyses, Local Authority identifiers were unavailable within the ELSA dataset, preventing adjustment for potential geographic clustering beyond repeated observations within individuals. As a result, we were unable to account for potential within-area clustering associated with these measures, which may have resulted in an underestimation of standard errors.

We acknowledge that standardized discrepancy scores do not distinguish between older adults who share the same discrepancy value but differ markedly in their absolute levels of loneliness or isolation (e.g., highly isolated versus minimally isolated older adults). Since this approach assumes commensurability of the two constructs after standardization, it captures relative divergence rather than absolute severity on either dimension. As a result, discrepancy scores which treat loneliness and isolation as equally weighted may partially reflect broader social patterning and socioeconomic gradients in loneliness or isolation rather than asymmetry per se. Residualized approaches offer an alternative but introduce their own limitations. By regressing loneliness on isolation (or vice versa), it frames social asymmetry as “excess loneliness” relative to predicted levels of isolation. This imposes a directional, sample-dependent interpretation, with residuals contingent on the strength and form of the association between the two variables. Given that loneliness and isolation were conceptualized here as related yet partially independent dimensions of social functioning, rather than as predictor and outcome, the standardized discrepancy approach aligns more closely with our theoretical framing of social asymmetry by directly indexing the degree to which subjective and objective social experiences diverge.

Although longitudinal and lagged analyses provided some insight into temporal ordering, causal inference remains limited. GEE models yield population-averaged associations and do not fully account for the unobserved heterogeneity or time-invariant confounding. Future research could extend this work by incorporating additional socioeconomic indicators, applying modeling approaches that disentangle between- and within-person effects, and examining trajectories of social asymmetry over longer follow-up periods. Such work may further clarify how socioeconomic conditions shape discrepancies between objective and subjective social experience across later life. We also recognize that several longitudinal panel studies of aging populations contain the necessary data to reconstruct and replicate our analyses. Future research would therefore benefit from coordinated cross-national panel modeling to assess the robustness of these associations across diverse contexts and to enhance the generalizability of our findings.

Overall, these findings suggest that social asymmetry in later life is not merely a reflection of individual psychological predispositions, but also a socially structured phenomenon. Discrepancies between objective social connectivity and subjective feelings of loneliness are patterned by socioeconomic stratification, highlighting who experiences social vulnerability and who demonstrates resilience. Understanding social asymmetry therefore requires attention to the material, perceptual, and contextual inequalities that shape how these social ties are experienced in later life. Efforts to reduce loneliness in older populations may need to move beyond individual-level interventions to address the broader socioeconomic inequalities that influence the alignment between social connectedness and felt belonging.

## Supplementary Material

gbag195_Supplementary_Data

## Data Availability

The data that support the findings are available upon registration with the UK Data Service, subject to standard checks: https://ukdataservice.ac.uk/. Code that supports the findings of this study is found in Supplementary Material. The study reported in this manuscript was not preregistered.
