# Supplementary material for: Socioeconomic correlates of social asymmetry in English older adults: findings from a population-based cohort study
Source: J Gerontol B Psychol Sci Soc Sci. 2026 Sep 15;81(11):gbag195. doi: 10.1093/geronb/gbag195 (PMC13718595; doi:10.1093/geronb/gbag195)
Supplement: Supplementary file 1 [file gbag195_supplementary_data.pdf]

The Journals of Gerontology, Series B: Psychological Sciences and Social Sciences
Supplementary Material: Calvey, Laurence, & McHugh Power. Socioeconomic correlates of social asymmetry in English older adults: Findings from a population-based cohort study.

Contents

Supplementary Table 1. Items which comprised measures of objective social isolation and subjective loneliness. 2

Supplementary Table 2. Time-lagged generalised estimating equation models where socioeconomic status indicators measured at time t predict social asymmetry at time t+1 (n=6586) 3

Supplementary Table 3. Weighted generalised estimating equation models using cross-sectional ELSA weights (n=8164)........ 4

Supplementary Table 4. Baseline descriptive characteristics of our sample at ELSA Wave 7 (n=6965), before and after imputation. 5

Supplementary Methods. R script for running generalised estimating equation models 7

Supplementary Table 1. Items which comprised measures of objective social isolation and subjective loneliness.

Modified Berkman-Syme Social Network Index (SNI)	*(Range: 0-4)*
Is married/cohabiting	Yes (=0); No (=1)
Has close friends	Yes (=0); No (=1)
Member in social or community groups	Yes (=0); No (=1)
Member in religious organisation*	Yes (=0); No (=1)
UCLA-3 Loneliness	*(Range: 0-6)*
How often respondent feels they lack companionship	Hardly ever or never (=0); Some of the time (=1); Often (=2)
How often respondent feels isolated from others	Hardly ever or never (=0); Some of the time (=1); Often (=2)
How often respondent feels in tune with the people around them	Hardly ever or never (=0); Some of the time (=1); Often (=2)

Note: * The original SNI assessed whether participants attended religious services.

Supplementary Table 2. Time-lagged generalised estimating equation models where socioeconomic status indicators measured at time t predict social asymmetry at time t+1 (n=6586)

	Estimate	SE	95% CI	p
Time	-.03	.02	(-.06 / .01)	.16
Age	-.01***	.002	(-.01 / -.01)	<.001
Sex: Female	-.05	.03	(-.11 / .01)	.11
Ethnicity: Non-white	-.11	.10	(-.30 / .08)	.25
Individual-level SES				
Education				
Lower Secondary	.20***	.05	(.10 / .29)	<.001
Upper Secondary	.35***	.06	(.23 / .47)	<.001
Third Level	.43***	.05	(.33 / .52)	<.001
Employment Status				
Employed	-.09*	.04	(-.16 / -.02)	.02
Unemployed	-.003	.05	(-.10 / .09)	.95
Subjective Social Status	.003**	.001	(.001 / .004)	.004
Individual Deprivation	-.03**	.01	(-.06 / -.01)	.002
Household Income				
Q2	.16***	.04	(.08 / .24)	<.001
Q3	.21***	.04	(.12 / .29)	<.001
Q4	.28***	.04	(.19 / .36)	<.001
Q5 (Highest)	.30***	.04	(.21 / .39)	<.001
Internet Use				
Sometimes	-.05	.04	(-.12 / .02)	.16
Rarely/Never	-.18***	.04	(-.26 / -.09)	<.001
Housing Tenure				
Private Renter	-.45***	.10	(-.65 / -.26)	<.001
Social Housing Renter	-.51***	.06	(-.63 / -.39)	<.001
Other	-.05	.13	(-.32 / .20)	.69
Area-level SES				
Area Deprivation				
Q2	-.10**	.03	(-.17 / -.04)	.002
Q3	-.12**	.04	(-.20 / -.05)	.002
Q4	-.09	.05	(-.18 / .01)	.06
Q5 (Highest)	-.24***	.06	(-.36 / -.12)	<.001
Population Density				
Q2	-.09*	.04	(-.17 / -.01)	.02
Q3	-.13**	.04	(-.22 / -.05)	.003
Q4	-.16***	.05	(-.25 / -.07)	<.001
Q5 (Greatest density)	-.14*	.06	(-.26 / -.03)	.01
Psychosocial Confounders				
Depressive Symptoms	-.11***	.01	(-.13 / -.09)	<.001
Self-rated Health	.07***	.01	(.04 / .10)	<.001
Satisfaction with Life	.04***	.003	(.04 / .05)	<.001

Notes: Significant at $*P < .05$, $**P < .01$, $***P < .001$; SE = standard errors; 95% CI = 95% Confidence Intervals. †Step 1 models were adjusted for time, age, sex and ethnicity, but estimates were not reported for these controls in Step 1. Reference categories: Sex: Male; Ethnicity: White; Education: None/Primary level; Employment status: Retired; Area Deprivation: Quintile 1 (Lowest Deprivation); Household Income: Quintile 1 (Lowest Earners); Population Density: Quintile 1 (Lowest density); Internet Use: Regularly; Housing Tenure: Homeowner.

Supplementary Table 3. Weighted generalised estimating equation models using cross-sectional ELSA weights (n=8164)

	Estimate	SE	95% CI	p
Time	-.04***	.01	(-.06 / -.02)	<.001
Age	-.002	.002	(-.007 / .002)	.33
Sex: Female	-.03	.03	(-.09 / .03)	.35
Ethnicity: Non-white	-.19	.11	(-.40 / .02)	.07
Individual-level SES				
Education				
Lower Secondary	.25***	.05	(.16 / .34)	<.001
Upper Secondary	.33***	.06	(.21 / .46)	<.001
Third Level	.44***	.05	(.35 / .53)	<.001
Employment Status				
Employed	-.11***	.03	(-.17 / -.04)	<.001
Unemployed	-.11*	.04	(-.19 / -.03)	.01
Subjective Social Status	.004***	.001	(.003 / .005)	<.001
Individual Deprivation	-.05***	.01	(-.07 / -.03)	<.001
Household Income				
Q2	.06	.04	(-.01 / .14)	.09
Q3	.17***	.04	(.09 / .25)	<.001
Q4	.19***	.04	(.10 / .26)	<.001
Q5 (Highest)	.25***	.04	(.16 / .33)	<.001
Internet Use				
Sometimes	-.06*	.03	(-.12 / -.002)	.04
Rarely/Never	-.28***	.04	(-.35 / -.21)	<.001
Housing Tenure				
Private Renter	-.29**	.09	(-.46 / -.11)	.001
Social Housing Renter	-.44***	.06	(-.55 / -.33)	<.001
Other	-.19	.12	(-.43 / .05)	.12
Area-level SES				
Area Deprivation				
Q2	-.04	.03	(-.11 / .02)	.18
Q3	-.06	.04	(-.14 / .01)	.10
Q4	-.07	.05	(-.16 / .02)	.12
Q5 (Highest)	-.19***	.06	(-.31 / -.08)	.001
Population Density				
Q2	-.05	.04	(-.12 / .03)	.19
Q3	-.09*	.04	(-.17 / -.01)	.02
Q4	-.10*	.04	(-.18 / -.01)	.02
Q5 (Greatest density)	-.15**	.05	(-.26 / -.05)	.003
Psychosocial Confounders				
Depressive Symptoms	-.13***	.01	(-.15 / -.11)	<.001
Self-rated Health	.03*	.01	(.002 / .05)	.03
Satisfaction with Life	.07***	.002	(.06 / .07)	<.001

Notes: Significant at $*P < .05$, $**P < .01$, $***P < .001$; SE = standard errors; 95% CI = 95% Confidence Intervals. †Step 1 models were adjusted for time, age, sex and ethnicity, but estimates were not reported for these controls in Step 1. Reference categories: Sex: Male; Ethnicity: White; Education: None/Primary level; Employment status: Retired; Area Deprivation: Quintile 1 (Lowest Deprivation); Household Income: Quintile 1 (Lowest Earners); Population Density: Quintile 1 (Lowest density); Internet Use: Regularly; Housing Tenure: Homeowner.

Supplementary Table 4. Baseline descriptive characteristics of our sample at ELSA Wave 7 (n=6965), before and after imputation.

	Before Imputation		After Imputation
	**% (n) / Mean (SD)**	**Missing (n)**	**% (n) / Mean (SD)**
**Age**	71.2 (8.30)	0	71.2 (8.30)
**Sex**		0	
Male	45.59% (n=3175)		45.59% (n=3175)
Female	54.41% (n=3790)		54.41% (n=3790)
**Ethnicity**		41	
White	97.05% (n=6720)		97.01% (n=6757)
Non-white	2.95% (n=204)		2.99% (n=208)
**Education**		30	
None/Primary	23.84% (n=1653)		23.82% (n=1659)
Lower secondary	31.53% (n=2187)		31.50% (n=2194)
Upper secondary	8.41% (n=583)		8.40% (n=585)
Third level	36.22% (n=2512)		36.28% (n=2527)
**Employment Status**		1	
Retired	74.34% (n=5177)		74.34% (n=5178)
Employed	17.73% (n=1235)		17.73% (n=1235)
Unemployed	7.93% (n=552)		7.93% (n=552)
**Subjective social status**	60.82 (16.9)	839	60.3 (17.1)
**Individual-level Deprivation**		3584	0.69 (1.81)
**Household Income**		664	
Q1	18.44% (n=1162)		17.96% (n=1251)
Q2	22.30% (n=1405)		21.75% (n=1515)
Q3	21.19% (n=1335)		21.18% (n=1475)
Q4	20.90% (n=1317)		21.32% (n=1485)
Q5 (Highest)	17.17% (n=1082)		17.79% (n=1239)
**Internet Use**		809	
Regularly	63.12% (n=3291)		51.68% (n=3600)
Sometimes	6.17% (n=1015)		16.20% (n=1128)
Rarely/Never	30.71% (n=1850)		32.12% (n=2237)
**Housing Tenure**		46	
Homeowner	84.98% (n=5879)		84.62% (n=5894)
Private Renter	2.46% (n=170)		2.56% (n=178)
Social Housing	11.34% (n=785)		11.34% (n=807)
Other	1.22% (n=85)		1.23% (n=86)
**Area-level Deprivation**		34	
Q1			25.99% (n=1810)
Q2			25.92% (n=1805)
Q3			21.11% (n=1470)
Q4			15.89% (n=1107)
Q5 (Highest)			11.10% (n=773)
**Population Density**		73	
Q1	21.33% (n=1470)		21.32% (n=1485)
Q2	23.61% (n=1627)		23.65% (n=1647)
Q3	22.43% (n=1546)		22.38% (n=1559)
Q4	20.23% (n=1394)		20.26% (n=1411)
Q5 (Greatest density)	12.40% (n=855)		12.39% (n=863)
**Depressive Symptoms**	1.23 (1.65)	52	1.23 (1.65)
**Self-rated Health**	3.31 (1.09)	3	3.31 (1.09)
**Satisfaction with Life**	22.91 (9.91)	0	22.91 (9.91)
**UCLA-3 Loneliness**			
Item 1	0.37 (0.59)	719	0.39 (0.60)
Item 2	0.34 (0.55)	760	0.36 (0.56)
Item 3	0.31 (0.54)	777	0.33 (0.56)
Total UCLA-3			1.07 (1.48)
**Modified SNI**			

	Before Imputation		After Imputation
	% (n) / Mean (SD)	Missing (n)	% (n) / Mean (SD)
Married/cohabiting	Yes (n=4752; 68.23%)<br>No (n=2213; 31.77%)	0	Yes (n=4752; 68.23%)<br>No (n=2213; 31.77%)
Has friends	Yes (n=5876; 93.76%)<br>No (n=403; 6.24%)	686	Yes (n=6484; 93.09%)<br>No (n=481; 6.91%)
Member in social group	Yes (n=4344; 72.24%)<br>No (n=1669; 27.76%)	952	Yes (n=4960; 71.21%)<br>No (n=2005; 28.79%)
Member in religious group	Yes (n=1281; 21.30%)<br>No (n=4732; 78.70%)	952	Yes (n=1478; 21.22%)<br>No (n=5487; 78.78%)
Total SNI			2.11 (0.68)

Supplementary Methods. R script for running generalised estimating equation models

```
# Standardised social asymmetry scores (wave 7 example)
# scale SNI
df$sni_wave7_standardised <- scale(df$sni_wave7, center = TRUE, scale = TRUE)

# scale UCLA
df$ucla7_standardised <- scale(df$ucla3_wave7, center = TRUE, scale = TRUE)

# subtract UCLA scores from SNI scores - discrepancy score
df$socialasymmetry_wave7 <- (df$sni_wave7_standardised -
df$ucla3_wave7_standardised)

# ensure discrepancy score is numeric
df$socialasymmetry_wave7 = as.numeric(df$socialasymmetry_wave7)

# Function to generate 95% confidence intervals for GEE models
confint.geeglm <- function(object, parm, level = 0.95, ...) {
  cc <- coef(summary(object))
  mult <- qnorm((1+level)/2)
  citab <- with(as.data.frame(cc),
              cbind(lwr=Estimate-mult*Std.err,
                   upr=Estimate+mult*Std.err))
  rownames(citab) <- rownames(cc)
  citab[parm,]
}

# Step 1 models
g1 <- geeglm(socialasymmetry ~ time + age + sex + ethnicity + educcation, id = idauniq,
data = df, family = gaussian, corstr = "exchangeable")
summary(g1)
confint(g1)

g2 <- geeglm(socialasymmetry ~ time + age + sex + ethnicity + employment , id = idauniq,
data = df, family = gaussian, corstr = "exchangeable")
summary(g2)
confint(g2)

g3 <- geeglm(socialasymmetry ~ time + age + sex + ethnicity + ind_deprivation, id = idauniq,
data = df, family = gaussian, corstr = "exchangeable")
summary(g3)
confint(g3)
```

g4 <- geeglm(socialasymmetry ~ time + age + sex + ethnicity + deprivation_quintiles, id = idauniq, data = df, family = gaussian, corstr = "exchangeable")
summary(g4)
confint(g4)

g5 <- geeglm(socialasymmetry ~ time + age + sex + ethnicity + income, id = idauniq, data = df, family = gaussian, corstr = "exchangeable")
summary(g5)
confint(g5)

g6 <- geeglm(socialasymmetry ~ time + age + sex + ethnicity + socialstatus, id = idauniq, data = df, family = gaussian, corstr = "exchangeable")
summary(g6)
confint(g6)

g7 <- geeglm(socialasymmetry ~ time + age + sex + ethnicity + density, id = idauniq, data = df, family = gaussian, corstr = "exchangeable")
summary(g7)
confint(g7)

g8 <- geeglm(socialasymmetry ~ time + age + sex + ethnicity + internet, id = idauniq, data = df, family = gaussian, corstr = "exchangeable")
summary(g8)
confint(g8)

g9 <- geeglm(socialasymmetry ~ time + age + sex + ethnicity + housing, id = idauniq, data = df, family = gaussian, corstr = "exchangeable")
summary(g9)
confint(g9)

# Step 2 model: Full SES model

g10 <- geeglm(socialasymmetry ~ time + age + sex + ethnicity + education + employment + ind_deprivation + deprivation_quintiles + income + socialstatus + density + internet + housing, id = idauniq, data = df, family = gaussian, corstr = "exchangeable")
summary(g10)
confint(g10)

# Step 3 model: Attenuation model

g11 <- geeglm(socialasymmetry ~ time + age + sex + ethnicity + education + employment + ind_deprivation + deprivation_quintiles + income + socialstatus + density + internet + housing + depression + srh + swl, id = idauniq, data = df, family = gaussian, corstr = "exchangeable")
summary(g11)

```
confint(g11)
```

# Sensitivity analysis: Time-lagged GEE

```
df2 <- df %>%
  arrange(idauniq, time) %>%
  group_by(idauniq) %>%
  mutate(socialasymmetry_lagged = lead(socialasymmetry)) %>%
  ungroup() %>%
  filter(!is.na(socialasymmetry_lagged)) %>%
  droplevels()

g12 <- geeglm(socialasymmetry_lagged ~ time + age + sex + ethnicity + education +
employment + ind_deprivation + deprivation_quintiles + income + socialstatus + density +
internet + housing + depression + srh + swl, id = idauniq, data = df2, family = gaussian,
corstr = "exchangeable")
summary(g12)
confint(g12)
```

# Sensitivity analysis: Weighted GEE

```
g13 <- geeglm(socialasymmetry ~ time + age + sex + ethnicity + education + employment +
ind_deprivation + deprivation_quintiles + income + socialstatus + density + internet +
housing + depression + srh + swl, id = idauniq, data = df, family = gaussian, corstr =
"exchangeable", weights = weight)
summary(g13)
confint(g13)
```
